# Dystrophic calcinosis: structural and morphological composition, and evaluation of ethylenediaminetetraacetic acid (‘EDTA’) for potential local treatment

**DOI:** 10.1186/s13075-024-03324-7

**Published:** 2024-05-22

**Authors:** Phillip Lee, Lorraine Green, Bartosz Marzec, Fiona Meldrum, Francesco Del Galdo, Begonya Alcacer-Pitarch

**Affiliations:** 1 School of Chemistry, University of Leeds, Woodhouse Lane, Leeds, LS2 9JT UK; 2 Leeds Institute of Rheumatic and Musculoskeletal Medicine, University of Leeds, Leeds, UK; 3 NIHR Leeds Musculoskeletal Biomedical Research Unit, Leeds Teaching Hospitals NHS Trust, Leeds, UK; 4 Leeds Teaching Hospitals Trust, Leeds, UK

**Keywords:** Calcinosis, Calcium deposits, EDTA treatment, Scleroderma, Systemic Sclerosis

## Abstract

**Background:**

To perform a detailed morphological analysis of the inorganic portion of two different clinical presentations of calcium-based deposits retrieved from subjects with SSc and identify a chemical dissolution of these deposits suitable for clinical use.

**Methods:**

Chemical analysis using Fourier Transform IR spectroscopy (‘FTIR’), Raman microscopy, Powder X-Ray Diffraction (‘PXRD’), and Transmission Electron Microscopy (‘TEM’) was undertaken of two distinct types of calcinosis deposits: paste and stone. Calcinosis sample titration with ethylenediaminetetraacetic acid (‘EDTA’) assessed the concentration at which the EDTA dissolved the calcinosis deposits in vitro.

**Results:**

FTIR spectra of the samples displayed peaks characteristic of hydroxyapatite, where signals attributable to the phosphate and carbonate ions were all identified. Polymorph characterization using Raman spectra were identical to a hydroxyapatite reference while the PXRD and electron diffraction patterns conclusively identified the mineral present as hydroxyapatite. TEM analysis showed differences of morphology between the samples. Rounded particles from stone samples were up to a few micron in size, while needle-like crystals from paste samples reached up to 0.5 µm in length.

Calcium phosphate deposits were effectively dissolved with 3% aqueous solutions of EDTA, in vitro. Complete dissolution of both types of deposit was achieved in approximately 30 min using a molar ratio of EDTA/HAp of ≈ 300.

**Conclusions:**

Stone and paste calcium-based deposits both comprise hydroxyapatite, but the constituent crystals vary in size and morphology. Hydroxyapatite is the only crystalline polymorph present in the SSc-related calcinosis deposits. Hydroxyapatite can be dissolved in vitro using a dosage of EDTA considered safe for clinical application. Further research is required to establish the optimal medium to develop the medical product, determine the protocol for clinical application, and to assess the effectiveness of EDTA for local treatment of dystrophic calcinosis.

## Background

Dystrophic calcinosis describes the deposition of calcium-containing crystals within the tissues in the presence of otherwise normal calcium and phosphate serum levels. It appears to occur in damaged/devitalised tissues, is often secondary to trauma or infection, and has been reported in autoimmune connective tissue diseases such as Systemic Sclerosis (SSc), Systemic Lupus Erythematosus (SLE) and Dermatomyositis (DM) [1, 2]. Although the pathogenesis of dystrophic calcinosis is not clear, it has been postulated that structural damage of tissues, age-related tissue changes, tissue hypoxia related to hypovascularity and genetical predisposition may play a role [2–4].

Histological analysis of calcinotic nodules reveals different types of nodular formations which vary in morphology, while sharing the same inorganic hydroxyapatite composition [5]. Clinically however, it is apparent that calcinosis can present in a range of manifestations, ranging from stone-like to granular paste/fluid-like deposits, and more importantly, with a variable degree of inflammation and related pain [4, 6, 7].

Particularly in patients with SSc, calcinosis can complicate the occurrence of digital ulcers or cause skin ulceration. In these cases, the presence of dystrophic calcinosis is associated with extreme pain, increased risk of infection and delayed healing of the skin ulceration. Independent of the pain, the affected tissues lose their function causing either a reduction in the range of motion of joint or muscle atrophy. The treatment of calcinosis is limited despite several attempts with various systemic and non-systemic approaches. Systematic reviews detail small case studies that have explored systemic management using calcium channel blockers, bisophosphonates, vitamin K antagonists, tetracyclines, anti-gout medication, cephalosporins, aluminium hydroxide, immunogobulins, biologic agents, antidotes and chelators [8, 9].

Early use of intravenous EDTA as a chelator has been explored with minimal to no benefit in juvenile cases of calcinosis presumed to be from dermatomyositis [10] despite contradicting success in several small case reports with SSc-related calcinosis, calciphalaxis and aortic calcification [11–13].

Unfortunately, the evidence on the efficacy of these systemic treatments is considered weak and their benefits are often short-lived due to re-occurrence of the dystrophic calcinosis.

Non-systemic treatments targeting the calcinosis deposit directly include surgical and scalpel debridement, laser therapy, extracorporeal shock wave lithotripsy, microdrilling, dry needling, intra-lesional sodium thiosulfate, intra-lesional steroid injections and topical ointment based preparations impregnated with neem oil or the chelator sodium thiosulfate [6, 14–23]. It has been suggested that these deposit-targeted treatments of calcinosis may have more favourable results than a systemic only approach [21]. This could be considered pertinent in SSc where blood supply and therefore penetration of medicaments to the soft tissues and extremities is compromised and therefore possibly less effective.

The aims of this study were to identify the composition of the inorganic components of the two clinical presentations of the calcium-based deposits (stone and paste) and to determine their efficacy of a potential therapeutic deposit targeted approach based on chemical dissolution with EDTA.

## Materials and methods

### Sample preparation

Twelve calcinosis samples were obtained from 6 patients with SSc attending the ulcer clinic at the Rheumatology Outpatients Department, Leeds Teaching Hospital NHS Trust (LTHT). The calcinosis samples were obtained from the wound beds of skin ulcers present on the upper and lower extremities; including lesions over the elbows, knees, ulnar shaft, shin, and fingers. Patients gave written informed consented to use the samples for research purposes. Ethical approval for the anlysis of routinely collected/discarded biosample was obtained (IRAS 178638).

The samples were separated into two groups according to their gross morphology: stone or paste. The stone group were round, solid deposits, typically a few millimeters in size. The paste group, consisted of dense fluid exudate, resembling a granular paste. The latter group has been previously described as calcic mousse and calcic pus mousse deposits (Amanzi et al. [24] clinical classification).

On extraction of the calcium deposits, they were immediately stored in a fridge at 4°C to prevent biological decomposition. Within 2 weeks the samples were sorted into their respective groups and submerged in 5% NaClO (Alfa Aesar, 11–14% available chlorine) for 24h, to remove all residual organic material left over from the surgical procedure. The samples were then vacuum filtered using a 0.22 μm Millipore GVHP01300 membrane, during which time ethanol and deionized water were alternatingly washed through to remove any microbes. During this process, all organic material previously oxidized with NaClO was filtered off and the application of ethanol further sterilized the samples. Finally, the samples were collected and in the case of the stone sample, the large particles were broken using an agate mortar and pestle to enable its composition to be determined.

### Composition analysis

The identities and morphologies of the mineral components of the deposits were investigated using four different methods: i) Fourier Transfer Infra-Red spectroscopy (‘FTIR’) and ii) Raman microscopy to obtain fingerprint spectra of the minerals present, iii) Powder X-Ray Diffraction (‘PXRD’) to determine the polymorphs of the crystalline materials present and iv) Transmission Electron Microscopy (‘TEM’) and associated Electron Diffraction (‘ED’) to determine the sizes, morphologies and polymorphs of the individual crystals present in both calcium-based deposits.

- i) FTIR Spectroscopy

FTIR spectroscopy is a non-destructive characterization technique, where absorption of infrared light results in molecular vibrations at characteristic wavelengths. Solid compounds exhibit fingerprint spectra that are identified by comparison with reference compounds. Each of the samples were analysed using a Perkin Elmer Spectrum 100 ATR-FTIR spectrometer (“ATR” is attenuated total reflection). A background scan with a 2 cm^−1^ resolution was used.

- ii) Raman Spectroscopy

Raman spectroscopy is used as a complementary technique to IR spectroscopy and can yield a fingerprint spectrum of solids due to light scattering rather than absorption. Raman spectra were recorded in the 1200 – 100 cm^−1^ range using a Renishaw inVia Raman Microscope equipped with a 785 nm laser, where the confocal microscope makes it possible to analyze individual particles (down to a few micron in size).

- iii) Powder X-ray Diffraction (‘PXRD’)

PXRD is an important analytical technique used to determine the structure of crystalline materials. A beam of X-rays is diffracted by a crystalline material, where the relationship between the angle of incidence of the X-rays with respect to the diffracting crystallographic planes and the inter-planar separation is described by Bragg’s Law. Samples for PXRD analysis ideally comprise a fine powder in which the individual crystallites are randomly oriented. The recorded patterns are then compared with databases of diffractograms of known substances or can be solved from first principles for new compounds.

The sample was dispersed in ethanol and pipetted onto an off-axis silicon wafer. The pattern was taken using a Bruker AXS D8 Advance P-XRD diffractometer. The X-ray lamp voltage was set to 40 kV and the current at 40 mA. The scan was conducted in the 4° – 70° 2θ range using 0.035 deg/s step size.

- iv) Transmission Electron Microscopy (‘TEM’)

TEM is an analytical and imaging technique that enables scientists to visualize solid samples at high magnifications, typically ranging between 5,000 to 350,000 ×. In contrast to traditional light microscopy, a TEM microscope utilizes an electron beam to produce an image of the specimen. Electron diffraction can simultaneously be performed in a TEM, and patterns can be recorded of small very quantities of material. In common with PXRD, this data can be used to determine the polymorph composition of the analyzed material.

The sample was dispersed in ethanol and pipetted onto a copper TEM grid and allowed to dry under ambient conditions. Transmission electron micrographs and electron diffraction patterns were obtained using a FEI Tecnai F20 microscope operated at 200 kV and equipped with a field emission gun and a Gatan CCD camera.

### Calcinosis sample titration with EDTA

A buffer composed of tris(hydroxymethyl)aminomethane and NaCl (‘TRIS’) whose pH was adjusted to 7.4 with 0.1 M NaOH was used as the titration solution. Its pH and ionic strength resembled that of a human plasma to allow the titration to proceed under physiological conditions. This consisted of 8.77 g of NaCl (Sigma Aldrich), 6.61 g of Tris–HCl (Sigma Aldrich) and 0.96 g of Tris-base (Sigma Aldrich) dissolved in deionized Milli-Q water. The pH of this mixture was then adjusted to 7.4 at 25 °C using NaOH solution (Fisher Chemical). The final product was labelled as physiological solution. Calcinosis deposits were later placed in this physiological solution and were titrated with EDTA.

Using a hot plate (Ika C-Mag HS7) and a contact thermometer (Ika ETS-D5), 50 ml of physiological solution in an Erlenmeyer flask was brought to 37 °C and stirred using a magnetic stirrer. Next, 100 mM EDTA (Thermo Scientific, disodium salt, dihydrate) was dissolved in the physiological solution and was used as the titrant. 3.8 mg of calcinosis sample was added to the Erlenmeyer flask and the magnetic stirrer positioned to enable the calcinosis sample to deposit on the bottom of the flask. This, coupled with a dark paper towel underneath the flask, facilitated detection of the titration end point. The burette was positioned within the neck of the Erlenmeyer flask and a seal was formed with parafilm to prevent evaporative loss of the physiological solution, whilst still allowing the titrant to be introduced. This process was repeated a minimum of six times for each of the two sample groups, which confirmed that the calcinosis dissolution with EDTA was reproducible and enabled us to carry out a statistical analysis on the obtained results.

## Results

### Composition analysis results

**Fig. 1:**
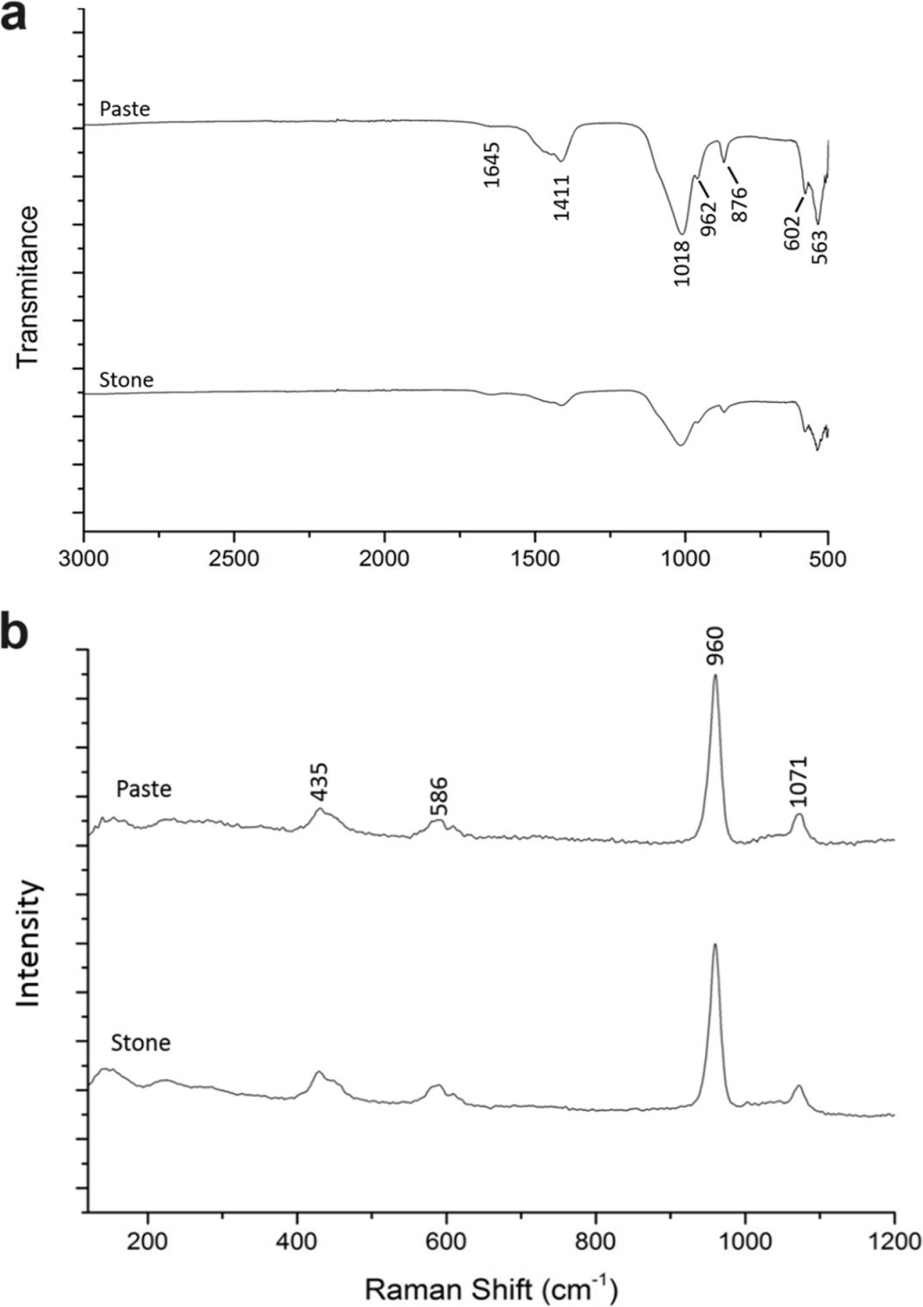
**a** FTIR spectra and (**b**) Raman spectra of paste and stone deposits, where both correspond to hydroxyapatite. The peaks in the Raman spectra correspond to the ν_3_ (1071 cm^−1^), ν_1_ (960 cm^−1^), ν_4_ (586 cm^−1^) and ν_2_ (435 cm^−1^) vibrational modes of phosphate ions present in the hydroxyapatite

Chemical analyses revealed that the deposits contained the widespread calcium phosphate biomineral, hydroxyapatite (HAp). The FTIR spectra for the samples are shown in Fig. 1a. The peak at 1018 cm^−1^ is characteristic of the phosphate ν_3_ stretching vibration for the apatite structure and indicates a poorly crystalline, non-stoichiometric apatite [25–27]. The peak at 962 cm^−1^ corresponds to the phosphate ν_1_ stretching vibration, while the peaks at 602 cm^−1^ and 563 cm^−1^ signify the phosphate ν_4_ bending mode [27, 28]. The FTIR spectra also exhibit peaks at 1645 cm^−1^, 1411 cm^−1^ and 876 cm^−1^, all of which can be attributed to carbonate ions. The carbonate ion may occupy two different positions within the HAp crystal lattice. A-type carbonated apatite involves a substitution at the OH^−^ site, whilst in B-type carbonated apatite, the phosphate ion is substituted. The latter of these is the major mineral species of biological apatites [29–31]. The lack of any other peaks indicated that no other chemical compound was present within the investigated samples. The Raman spectra also confirm that the samples are hydroxyapatite (Fig. 1b). The peaks at 1071 cm^−1^, 960 cm^−1^, 586 cm^−1^ and 435 cm^−1^ each correspond to the ν_3_, ν_1_, ν_4_ and ν_2_ vibrational modes of the phosphate ion, respectively [25, 29, 32].

**Fig. 2:**
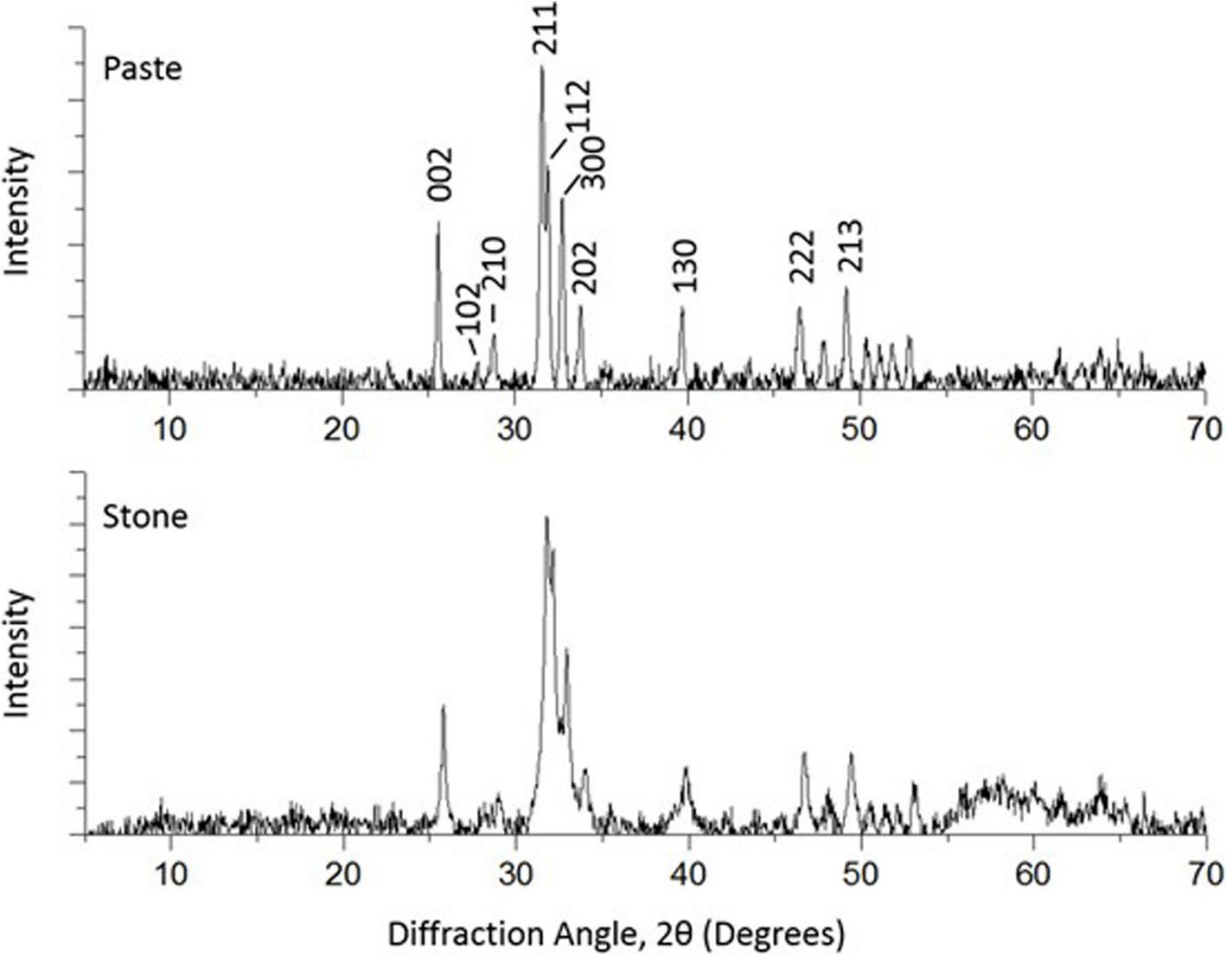
Powder XRD patterns of paste (top) and stone (bottom) calcium deposits. Both graphs show the same positions of the reflections, which are indexed for hydroxyapatite. These results confirm that the paste and stone deposits both contain hydroxyapatite

Powder XRD was used to conclusively identify the crystal polymorph (Fig. 2). The PXRD patterns indicate that the samples are apatitic in structure [27, 28, 33–35] and both types of samples exhibited identical sets of reflections. The positions of the observed reflections were consistent with the theoretical positions of signals produced by hydroxyapatite, which confirmed that despite their different appearances, both samples contained the same crystalline inorganic material.

### Crystal sizes and morphologies

**Fig. 3:**
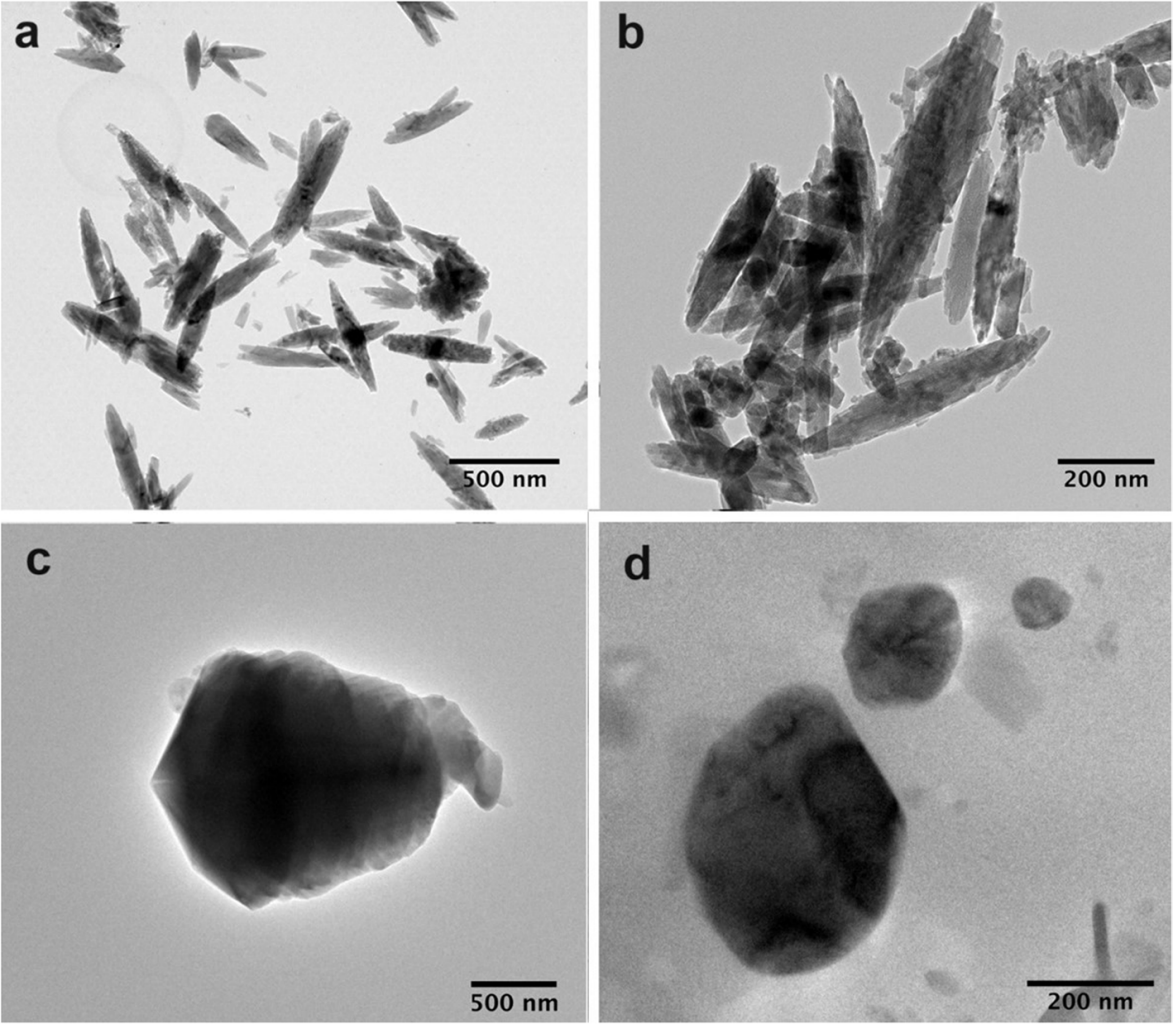
Transmission electron micrographs of paste calcinosis (**a** & **b**) and stone calcinosis (**c** & **d**) particulates. The recorded images reveal the difference in morphologies of the two samples. The paste sample contained needle-like crystals with tapered ends while the stone sample is composed of rounded particles. The hydroxyapatite crystals in the deposits therefore display very different morphologies, where this may be indicative of different stages of the calcium deposit formation

Transmission Electron Microscopy (TEM) was utilised to investigate the crystal structure, size and morphology of the two types of calcinosis deposits investigated here (Fig. 3). The micrographs reveal differences in the sizes and morphologies. Stone samples contained rounded hydroxyapatite crystals that often reached microns in size, while those in the paste sample possessed acicular morphologies and were typiclly sub-micron in size. The latter shape is typical of HAp formed in the presence of organic growth modifiers [36–39]. The differences in the crystal morphologies is also reflected in the different relative intensities of the peaks in the PXRD patterns, where the acicular particles will exhibit preferred orientation on the silicon wafer substrate (lying flat, as opposed to end-on).

**Fig. 4:**
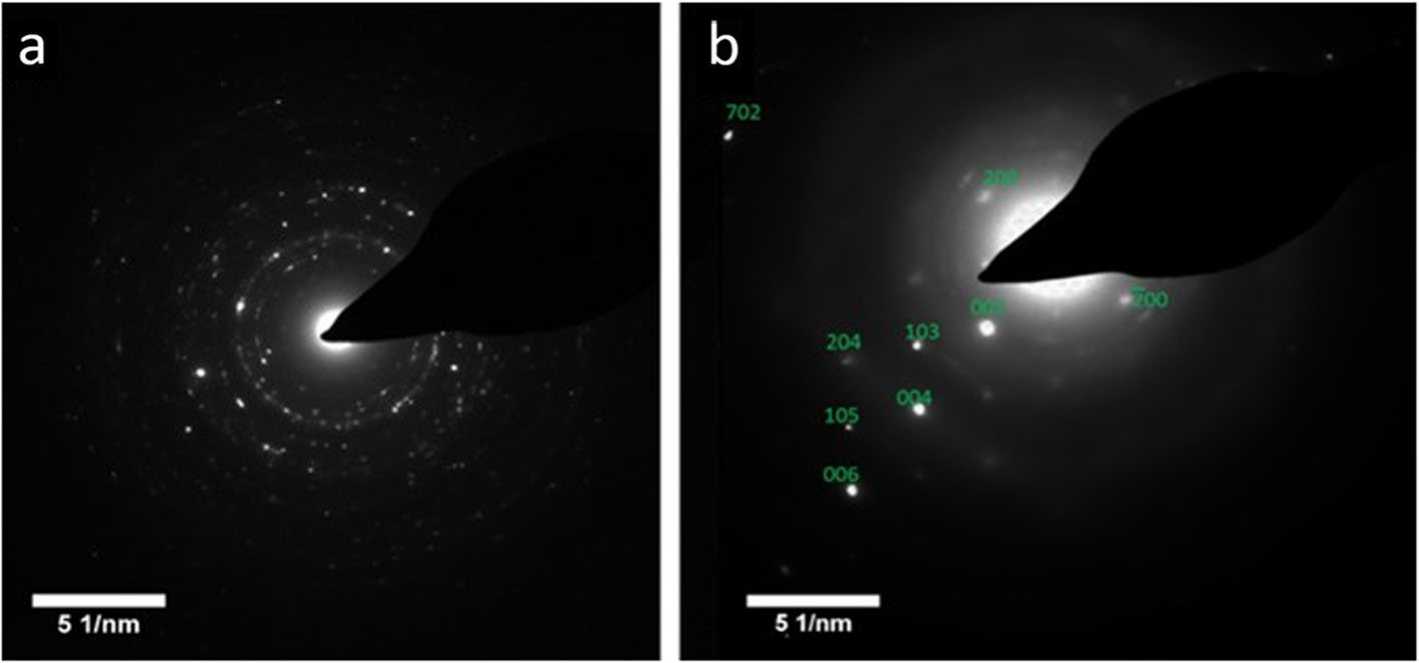
Electron diffraction patterns of (**a**) the paste sample, and (**b**) the stone sample, indicating that both are crystalline hydroxyapatite. The contribution of a large single crystal to the pattern can be seen in (**b**), where the distinct reflections are indexed

**Fig. 5:**
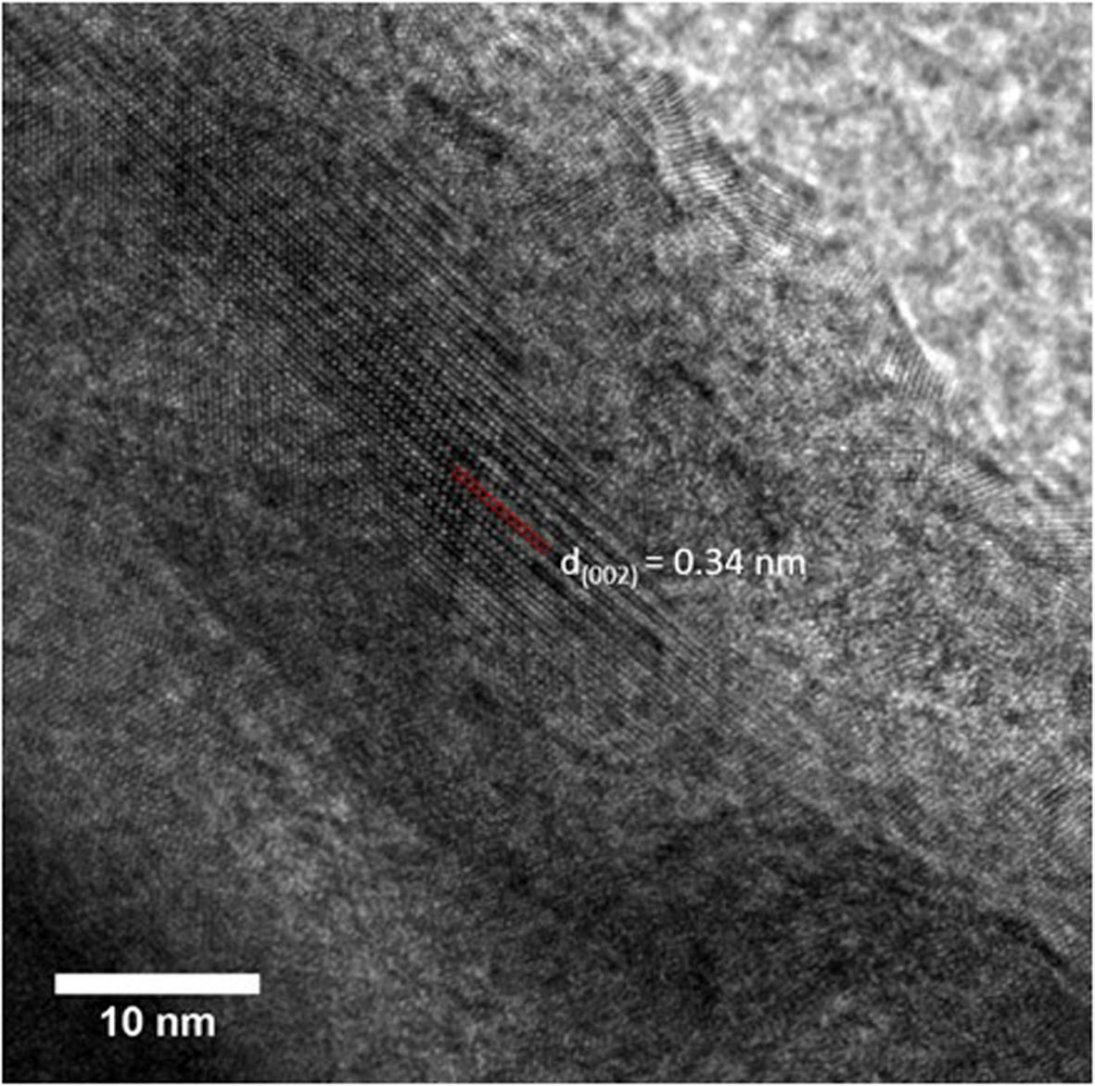
High-resolution TEM micrograph of a hydroxyapatite particle from a stone sample showing lattice fringes. The lattice plane spacing of 0.34 nm corresponds to the (002)-plane in Hap

Electron diffraction (ED) patterns of the calcinosis samples further confirmed that both were crystalline HAp. Spotty rings, as seen in the paste sample electron diffraction pattern (Fig. 4a) are associated with polycrystalline agglomerates where the individual crystallites exhibit different orientations on the TEM grid. That rings were clearer in the ED patterns of the paste sample is consistent with the smaller size of the crystallites, such that more are sampled when the pattern is recorded. ED from the stone samples yielded an ordered pattern consistent with a larger single crystal, together with additional bright spots arising from further crystallites (Fig. 4b). High Resolution-TEM was also used to investigate a stone sample, and the obtained lattice images revealed a spacing of 0.34 nm that corresponds to the (002)-plane in HAp (Fig. 5) [40].

### Calcinosis sample titration with EDTA results

**Fig. 6:**
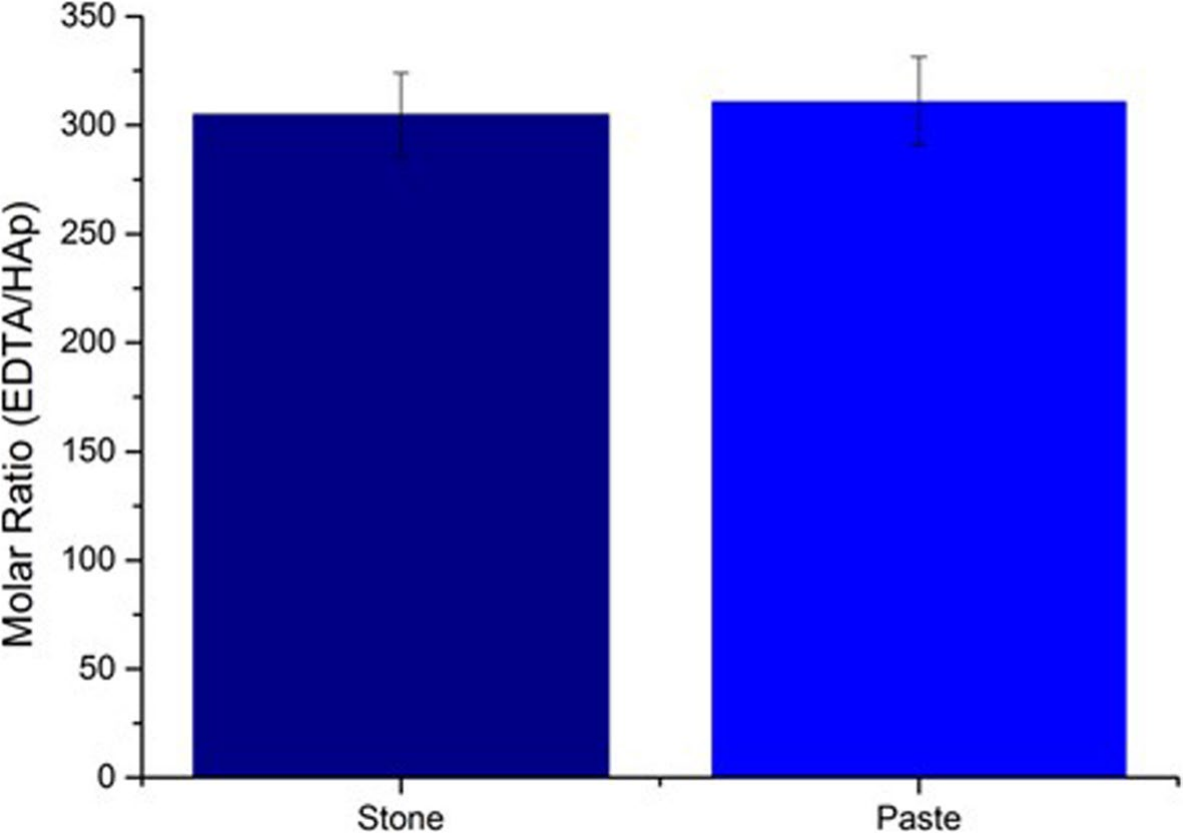
Molar ratio of EDTA: HAp required to dissolve the stone and paste sample of calcinosis cutis in 30 min. The EDT:HAp (± std. error) for the stone is 305 (± 19) and for the paste 311 (± 20), demonstrating the same solubility. Complete dissolution of the deposits was achieved in 30 min under these conditions

Dissolution of the calcinosis samples was investigated by titrating the stone and paste samples with an EDTA solution. Both the calcinosis sample and EDTA were prepared in solutions containing NaCl, and TRIS buffer adjusted to pH 7.4 to emulate the physiological conditions of human plasma. Using the end-point of the titration, it was possible to calculate the EDTA:HAp molar ratio required to completely dissolve the calcinosis deposits (Fig. 6). The molar ratios of EDTA/HAp (± std. error) required to completely dissolve the stone and paste samples in 30 min were 305 (± 19) and 311 (± 20) respectively, showing that both behaved similarly. The pH of the solution determines the efficacy of EDTA as a chelating agent, where the pH of the solution used here was 7.4. At high pH, a greater number of ionised EDTA species exist in solution (the active form), though the HAp dissolution rate is reduced as it is less soluble at high pH. At low pH the HAp dissolved more readily, but the EDTA chelates Ca^2+^ ions less effectively as the functional groups become protonated [41]. Hence, the optimum pH for EDTA to dimineralize HAp is between pH 6 and 10 [42].

## Discussion

The aims of this study were to identify the morphology of the mineral within two different clinical presentation of calcium-containing deposits (stone and paste), and to determine whether future therapeutic approaches can be developed based on chemical dissolution of the deposits.

Our results show that both types of deposit contain the same crystal phase i.e. hydroxyapatite. Previous literature has examined the chemical compositions of various skin calcified deposits by vibrational spectroscopy describing different crystalline polymorphs of Calcium phosphate (CaP). Within the reports of calcium deposits examined from SSc subjects, type B hydroxyapatite carbonate, scattered apatite crystals and interglobular apatite have been described [43]. A later study using x-ray diffraction confirmed that calcium deposits in SSc are composed of calcium hydroxyapatite with most samples containing more than 50% of other organic material [44]. From a medical perspective, hydroxyapatite is the most important CaP polymorph, as it is used by a human body to produce hard tissues, including bones and teeth. Deposition of hydroxyapatite in vivo as bones and teeth is a genetically controlled and evolution refined process that occurs within controlled microenvironments. However, in dystrophic calcinosis, this refined process becomes abnormal and the deposition of CaP is misplaced in different areas of the body. These appear to be areas of damaged and devitalised tissues, and calcinosis is often secondary to trauma or infection.

While the chemical composition of the stone and paste calcium-based deposits is the same, the morphology of the constituent crystalline particles is different, namely round hydroxyapatite deposits for the stone type and needle-like particles for the paste type. The difference in morphology might also influence soft-tissue inflammation around the calcium deposits, a common clinical occurrence, although clinical characteristics of the lesions were not assessed in this study. Prudhommeaux et al. investigated the effect of hydroxyapatite ultrastructure and physicochemical composition on the inflammatory response [45]. They concluded that, aside from stoichiometric HAp, the greatest inflammatory response was caused by carbonate-substituted apatite. A greater effect was also observed with increasing specific surface areas (SS) of the HAp crystals up to 50 m^2^ g^−1^, consistent with a response to the surface of the crystals. Approximating the HAp crystals in the stone sample to spheres of radii 1000 nm and those in the paste sample to cylinders of dimensions 500 nm × 100 nm, and taking a density of HAp of 3.16 g cm^−3^, the calculation gives SS values of 9.48 m^2^ g^−1^ and 31.6 m^2^ g^−1^ for the stone and paste samples respectively. A greater inflammatory response is therefore predicted to the crystals in the the paste sample. Research has additionally revealed that biogenic HAp crystals have atomically rough surfaces, which provides them with the propensity to bind to proteins and interact with cell membranes [46]. These findings suggest that of the two sample types, the paste sample may be associated with greater inflammatory response.

In addition, the paste sample might represent a different time-point in the disease progression than the stone sample. In calcific tendonitis, a related form of calcinosis, the maturity of a deposit can be determined by its crystallinity, which progressively increases as the deposit matures [47]. We can hypothesize that the development of a calcinosis deposit starts with the precipitation of small, needle-shaped crystals (as observed in the paste samples), and eventually progress to the larger, rounded particles seen in the stone samples. This could conceivably occur via an aggregation/ recrystallisation mechanism, although the exact mechanism remains unclear and requires further investigation.

The next stage in the study was to evaluate a potential treatment for dystrophic calcinosis. Our study shows that the chelating agent EDTA was remarkably effective in rapidly dissolving both types of calcium-containing deposits in vitro. This agent was chosen due to its high ability to chelate Ca^2+^ cations, its low toxicity at moderate concentrations and its current use in clinical settings. EDTA is used in chelation therapy for heavy metal poisoning [48] and is also widely used in dentistry to aid in the removal of the inorganic contribution of the smear layer [49–55]. The elimination of the smear layer, an inorganic/organic composite film formed as a result of instrumentation during root canal treatment [56] is a necessary step to prevent infection and to allow the penetration of intracanal medication to the dentine tubules [56]. In this instance, EDTA is combined with sodium hypochlorite, which acts as a disinfectant. The concentration of EDTA in these solutions is between 17 and 25% [49–55, 57–60]. When used in cosmetics it is 2% and the lowest dose reported to cause a toxic effect in animals while administered by injection was 750 mg/kg/day [61]. Considering that the EDTA solution used here was effective at 3 m%, the use of EDTA as a treatment for calcinosis cutis is promising.

In this in vitro experiment, it was also observed that chemical dissolution of the calcium-based deposits has the potential to remove the inorganic material from the calcinotic lesions present in a patient. Treatment with EDTA solutions therefore has the potential to offer a clinical advantage over other currently available treatment procedures, which rely on the mechanical removal of the deposits and typically leave residual crystals in the affected tissue. The hydroxyapatite particles left in the wound can then act as seeds for further hydroxyapatite deposition, such that the formation of new deposits is promoted at the same location. Therefore, the application of EDTA solutions on the wound bed of a skin ulcer caused by calcium deposits or on a wound bed following a surgical extraction of calcium deposits has the potential to remove any residual crystals in the affected tissue, preventing the formation of new deposits and aiding healing. However, further in vivo research and clinical trials are needed to confirm these hypotheses.

It is important to highlight that the calcium deposits samples were obtained only from patients with SSc, and no samples were obtained from individuals with other rheumatic and musculoskeletal diseases. All organic material was removed and deposits crushed prior to dissolution of the crytals using EDTA which is not representative of what can be achieved in the clinical setting, although it has been suggested that the effect of EDTA is also related to its ability to dissolve organic material too. Further studies in vitro preserving the surrounding organic material to represent the clinical setting and later studies applying a preparation of EDTA in vivo are required to assess the full potential of EDTA as a safe and effective treatment of calcinosis.

## Conclusion

Our results show that the stone and paste calcium-containing deposits both comprise carbonate-substituted biogenic hydroxyapatite. However, the crystals in each of these deposits are quite distinct in size and morphology. While the stone samples are composed of rounded crystals up to a few microns in diameter, the paste samples are acicular in shape and ≈ 0.5 µm in length. We also demonstrate that the chelating agent EDTA has the potential to be used to develop an effective medical product for the local treatment of dystrophic calcinosis. 100 mM EDTA with an acting time of 30 min was sufficient to cause complete dissolution of both stone and paste materials in vitro. Considering that this concentration falls within currently accepted safe limits of EDTA exposure, this agent has the potential to be used in medical products for local treatment of calcium deposits present in wound-care, potentially dissolving and preventing further formation of calcium-based deposits. Future research is required to establish the optimal medium to develop the medical product, and define a protocol for clinical application. This will then inform a randomized controlled trial to assess the effectiveness of EDTA for local treatment of dystrophic calcinosis.

## Data Availability

No datasets were generated or analysed during the current study.

## Abbreviations

ATR: Attenuated Total Reflection
CaP: Calcium phosphate
DM: Dermatomyositis
ED: Electron Diffraction
EDTA: Ethylenediaminetetraacetic Acid
FTIR: Fourier Transform IR spectroscopy
Hap: Hydroxyapatite
LTHT: Leeds Teaching Hospital NHS Trust
NaCl: Sodium chloride
NaClO: Sodium hypochlorite
NaOH: Sodium hydroxide
OH^−^: Hydroxide ion
pH: Potential of Hydrogen
PXRD: Powder X-Ray Diffraction
SLE: Systemic Lupus Erythematosus
SS: Specific Surface Areas
SSc: Systemic Sclerosis
TEM: Transmission Electron Microscopy
TRIS: Tris(hydroxymethyl)aminomethane and NaCl
